# *tert*-Butyl­dimethyl­silanol hemihydrate

**DOI:** 10.1107/S1600536808015444

**Published:** 2008-05-30

**Authors:** Sarah M. Barry, Helge Mueller-Bunz, Peter J. Rutledge

**Affiliations:** a Centre for Synthesis and Chemical Biology, University College Dublin, Belfield, Dublin 4, Ireland; b School of Chemistry and Chemical Biology, University College Dublin, Belfield, Dublin 4, Ireland; c School of Chemistry F11, University of Sydney, NSW 2006, Australia

## Abstract

The crystal structure of the title compound, C_6_H_16_OSi·0.5H_2_O, reveals an asymmetric unit containing two mol­ecules of the silanol and a single water mol­ecule. There is evidence of hydrogen bonding between the three mol­ecules in the asymmetric unit. The H atoms of the silanol OH groups and the water H atoms are each disordered equally over two positions.

## Related literature

For related literature, see: Krall *et al.* (2005 ▶); Lickiss *et al.* (1995 ▶); Mansfeld, Schürmann & Mehring (2005 ▶); Mansfeld, Mehring & Schürmann (2005 ▶); McGeary *et al.* (1991 ▶); Veith *et al.* (2006 ▶); Barry & Rutledge (2008 ▶); Görbitz (1999 ▶).
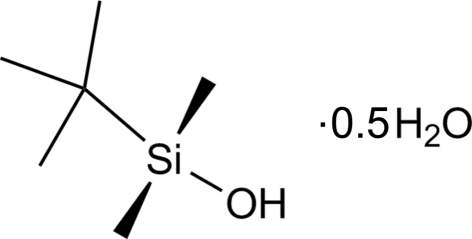


## Experimental

### 

#### Crystal data

C_6_H_16_OSi·0.5H_2_O
*M* *_r_* = 141.29
Monoclinic, 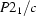

*a* = 7.7078 (18) Å
*b* = 22.119 (5) Å
*c* = 11.058 (3) Å
β = 90.307 (4)°
*V* = 1885.2 (8) Å^3^
*Z* = 8
Mo *K*α radiation
μ = 0.19 mm^−1^
*T* = 100 (2) K
1.00 × 1.00 × 0.80 mm

#### Data collection

Bruker SMART APEX detector diffractometer
Absorption correction: multi-scan (*SADABS*; Sheldrick, 2000 ▶) *T*_min_ = 0.519, *T*_max_ = 0.865
15971 measured reflections
4093 independent reflections
3529 reflections with *I* > 2σ(*I*)
*R*_int_ = 0.054

#### Refinement

*R*[*F* ^2^ > 2σ(*F* ^2^)] = 0.055
*wR*(*F* ^2^) = 0.149
*S* = 1.05
4093 reflections
181 parameters
6 restraints
H atoms treated by a mixture of independent and constrained refinement
Δρ_max_ = 0.55 e Å^−3^
Δρ_min_ = −0.49 e Å^−3^

### 

Data collection: *SMART* (Bruker, 2001 ▶); cell refinement: *SAINT* (Bruker, 2001 ▶); data reduction: *SAINT*; program(s) used to solve structure: *SHELXS97* (Sheldrick, 2008 ▶); program(s) used to refine structure: *SHELXL97* (Sheldrick, 2008 ▶); molecular graphics: *SHELXTL* (Sheldrick, 2008 ▶); software used to prepare material for publication: *SHELXTL*.

## Supplementary Material

Crystal structure: contains datablocks I, global. DOI: 10.1107/S1600536808015444/kj2088sup1.cif

Structure factors: contains datablocks I. DOI: 10.1107/S1600536808015444/kj2088Isup2.hkl

Additional supplementary materials:  crystallographic information; 3D view; checkCIF report

## Figures and Tables

**Table 1:** Hydrogen-bond geometry (Å, °)

*D*—H⋯*A*	*D*—H	H⋯*A*	*D*⋯*A*	*D*—H⋯*A*
O1—H1*O*1⋯O3^i^	0.84	2.09	2.717 (3)	131
O1—H2*O*1⋯O3	0.84	1.96	2.706 (3)	147
O2—H1*O*2⋯O3^ii^	0.84	2.04	2.718 (3)	138
O2—H2*O*2⋯O3	0.84	2.05	2.707 (3)	135
O3—H1*O*3⋯O1	0.824 (19)	1.91 (3)	2.706 (3)	163 (6)
O3—H4*O*3⋯O1^i^	0.815 (19)	1.92 (2)	2.717 (3)	164 (6)
O3—H2*O*3⋯O2	0.82 (2)	1.89 (2)	2.707 (3)	173 (6)
O3—H3*O*3⋯O2^ii^	0.822 (19)	1.92 (2)	2.718 (3)	164 (6)

Symmetry codes: (i) 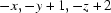; (ii) 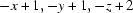.

**Fig. 1.**
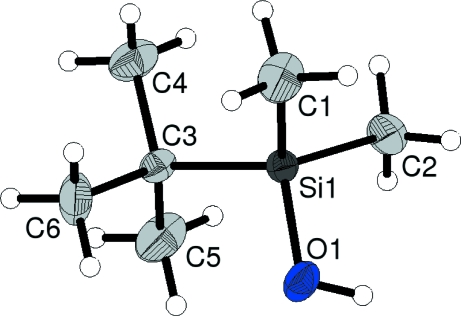
The molecular structure of the title compound with atom labels and thermal ellipsoids drawn on the 50% probability level. Carbon atoms are shown in grey, the silicon in black and the oxygen in blue.

**Fig. 2.**
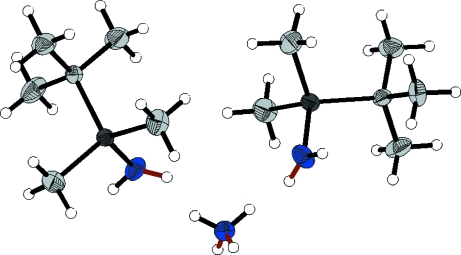
View of two adjacent silanols and a bridging water showing the disorder that is present (Part 1 black bonds, Part 2 brown bonds); thermal ellipsoids are drawn on the 50% probabibility level.

## supplementary crystallographic information

### Comment

The stucture of the title compound *tert*-butyldimethylsilanol hemihydrate is shown below (Fig. 1, 2); dimensions are available in the archived CIF. This compound has previously been characterized by gas-phase electron diffraction of both the free silanol and its hemihydrate (Lickiss *et al.*,  1995). It has also been structurally characterized within lanthanoid complexes (McGeary *et al.*,  1991) and in recent structures of complexes with several main group and transition metals (for examples see Mansfeld, Mehring & Schürmann, 2005; Mansfeld, Schürmann & Mehring, 2005, Veith *et al.*, 2006). However direct crystallographic characterization of the silanol has hitherto remained elusive.

*tert*-Butyldimethylsilanol hemihydrate was isolated in crystalline form during the synthesis of biomimetic ligands for iron-mediated hydrocarbon oxidation (Krall *et al.*,  2005, Barry & Rutledge, 2008). The title compound was obtained as the side product of reactions to prepare a compound incorporating the *tert*-butyldimethylsilyl ether as a protecting group (2-(bromomethyl)-6-((*tert*-butyldimethylsilyloxy)methyl)pyridine).

The assymetric unit contains two molecules of the silanol and one water molecule, linked by hydrogen bonding. The hydrogen atoms of the water molecule and the silanol O—H groups are disordered between two alternative occupancies (Fig. 2).

### Experimental

The title compound crystallized serendipitously as colourless needles from a sample of the silyl ether 2-(bromomethyl)-6-((*tert*-butyldimethylsilyloxy)methyl)pyridine (isolated as an oil from a pentane:ether solvent mixture), upon standing at room temperature for several weeks.

Large crystals of the silanol were obtained (1.00 × 1.00 × 0.80 mm) and data was collected from a crystal at the upper size limit of the beam used. Moreover, the crystals of *tert*-butyldimethylsilanol hemihydrate that have been isolated exhibit remarkably low density (0.996 g cm^-3^), a property that may be linked to the high volitility of this compound.

### Refinement

A full sphere of the reciprocal space was scanned by φ-ω scans. Hydrogen atoms of the silanol molecules were added at calculated positions and refined using a riding model. C–H distances were assumed to be 0.98 Å, O–H distances to be 0.84 Å. The water protons were located in the difference Fourier map. The distance of these protons to the oxygen atom was restrained to be 0.84 Å using the *DFIX* command. In the same way the H–O–H angles were restrained to be 114°, the value to which a preliminary refinement of one component converged. U_iso_(H) = 1.5 U_eq_(carrier) for all H atoms.

The site occupation factor of the disordered hydrogen atoms was fixed to 0.5. Attempts to refine the occupation factors were unsuccessful. However, electron densities in the difference Fourier map suggest a fairly even distribution between the two disordered parts.

Discrepancies between the expected and reported values of the maximum and minimum transmission (T_max_/T_min_) are thought to have arisen from the large size of the crystal relative to the beam, and because the crystal mount has given rise to some absorption during data collection. However this is not thought to impact significantly on the dataset given that there is an almost fourfold redundancy with the collection of a full sphere, and the capacity of *SADABS* to handle data collected from large crystals (Görbitz, 1999, Sheldrick, 2000).

### Crystal data

**Table:**

C_6_H_16_OSi·0.5H_2_O	*F*_000_ = 632
*M**_r_* = 141.29	*D*_x_ = 0.996 Mg m^−^^3^
Monoclinic, *P*2_1_/*c*	Mo *K*α radiation λ = 0.71073 Å
*a* = 7.7078 (18) Å	Cell parameters from 5333 reflections
*b* = 22.119 (5) Å	θ = 2.6–29.4º
*c* = 11.058 (3) Å	µ = 0.19 mm^−^^1^
β = 90.307 (4)º	*T* = 100 (2) K
*V* = 1885.2 (8) Å^3^	Block, colourless
*Z* = 8	1.00 × 1.00 × 0.80 mm

### Data collection

**Table:**

Bruker SMART APEX detector diffractometer	4093 independent reflections
Radiation source: fine-focus sealed tube	3529 reflections with *I* > 2σ(*I*)
Monochromator: graphite	*R*_int_ = 0.054
*T* = 100(2) K	θ_max_ = 27.0º
φ and ω scans	θ_min_ = 0.9º
Absorption correction: multi-scan(SADABS; Sheldrick, 2000)	*h* = −9→9
*T*_min_ = 0.519, *T*_max_ = 0.865	*k* = −28→28
15971 measured reflections	*l* = −14→14

### Refinement

**Table:**

Refinement on *F*^2^	Secondary atom site location: difference Fourier map
Least-squares matrix: full	Hydrogen site location: inferred from neighbouring sites
*R*[*F*^2^ > 2σ(*F*^2^)] = 0.055	H atoms treated by a mixture of independent and constrained refinement
*wR*(*F*^2^) = 0.149	*w* = 1/[σ^2^(*F*_o_^2^) + (0.0859*P*)^2^ + 1.0972*P*] where *P* = (*F*_o_^2^ + 2*F*_c_^2^)/3
*S* = 1.05	(Δ/σ)_max_ = 0.040
4093 reflections	Δρ_max_ = 0.55 e Å^−^^3^
181 parameters	Δρ_min_ = −0.49 e Å^−^^3^
6 restraints	Extinction correction: none
Primary atom site location: structure-invariant direct methods	

### Special details

**Table:**

Geometry. All e.s.d.'s (except the e.s.d. in the dihedral angle between two l.s. planes) are estimated using the full covariance matrix. The cell e.s.d.'s are taken into account individually in the estimation of e.s.d.'s in distances, angles and torsion angles; correlations between e.s.d.'s in cell parameters are only used when they are defined by crystal symmetry. An approximate (isotropic) treatment of cell e.s.d.'s is used for estimating e.s.d.'s involving l.s. planes.
Refinement. Refinement of *F*^2^ against ALL reflections. The weighted *R*-factor *wR* and goodness of fit *S* are based on *F*^2^, conventional *R*-factors *R* are based on *F*, with *F* set to zero for negative *F*^2^. The threshold expression of *F*^2^ > σ(*F*^2^) is used only for calculating *R*-factors(gt) *etc*. and is not relevant to the choice of reflections for refinement. *R*-factors based on *F*^2^ are statistically about twice as large as those based on *F*, and *R*- factors based on ALL data will be even larger.

### Fractional atomic coordinates and isotropic or equivalent isotropic displacement parameters (Å^2^)

**Table:**

	*x*	*y*	*z*	*U*_iso_*/*U*_eq_	Occ. (<1)
Si1	0.08637 (10)	0.59717 (3)	1.24126 (7)	0.01975 (18)	
O1	0.0393 (3)	0.56062 (9)	1.1151 (2)	0.0313 (5)	
H1O1	−0.0509	0.5399	1.1251	0.047*	0.50
H2O1	0.1314	0.5488	1.0825	0.047*	0.50
C1	0.0225 (5)	0.55098 (15)	1.3734 (3)	0.0408 (8)	
H1A	−0.1001	0.5398	1.3660	0.061*	
H1B	0.0400	0.5744	1.4477	0.061*	
H1C	0.0937	0.5143	1.3766	0.061*	
C2	0.3243 (4)	0.60908 (15)	1.2437 (3)	0.0350 (7)	
H2A	0.3834	0.5699	1.2401	0.052*	
H2B	0.3575	0.6301	1.3184	0.052*	
H2C	0.3580	0.6336	1.1738	0.052*	
C3	−0.0367 (4)	0.67096 (12)	1.2379 (2)	0.0227 (5)	
C4	−0.0021 (5)	0.70679 (15)	1.3542 (3)	0.0385 (8)	
H4A	0.1224	0.7151	1.3616	0.058*	
H4B	−0.0405	0.6831	1.4240	0.058*	
H4C	−0.0661	0.7451	1.3512	0.058*	
C5	0.0191 (6)	0.70852 (15)	1.1297 (3)	0.0471 (10)	
H5A	−0.0453	0.7467	1.1288	0.071*	
H5B	−0.0051	0.6861	1.0550	0.071*	
H5C	0.1437	0.7169	1.1355	0.071*	
C6	−0.2315 (4)	0.65768 (16)	1.2285 (3)	0.0425 (8)	
H6A	−0.2677	0.6336	1.2984	0.064*	
H6B	−0.2553	0.6351	1.1541	0.064*	
H6C	−0.2961	0.6958	1.2271	0.064*	
Si2	0.41635 (10)	0.62186 (3)	0.80713 (7)	0.02124 (19)	
O2	0.4716 (3)	0.56071 (10)	0.8832 (2)	0.0345 (5)	
H1O2	0.5780	0.5619	0.8996	0.052*	0.50
H2O2	0.3931	0.5346	0.8761	0.052*	0.50
C7	0.4726 (5)	0.68968 (15)	0.8981 (3)	0.0402 (8)	
H7A	0.3994	0.6911	0.9703	0.060*	
H7B	0.4531	0.7261	0.8496	0.060*	
H7C	0.5948	0.6876	0.9225	0.060*	
C8	0.1783 (4)	0.61756 (15)	0.7803 (3)	0.0381 (8)	
H8A	0.1509	0.5805	0.7356	0.057*	
H8B	0.1407	0.6528	0.7332	0.057*	
H8C	0.1179	0.6172	0.8581	0.057*	
C9	0.5397 (4)	0.62185 (11)	0.6618 (2)	0.0226 (6)	
C10	0.4979 (5)	0.56497 (14)	0.5889 (3)	0.0379 (8)	
H10A	0.5657	0.5650	0.5142	0.057*	
H10B	0.3739	0.5643	0.5690	0.057*	
H10C	0.5274	0.5291	0.6369	0.057*	
C11	0.7347 (4)	0.62329 (15)	0.6898 (3)	0.0375 (8)	
H11A	0.7654	0.5886	0.7407	0.056*	
H11B	0.7634	0.6608	0.7325	0.056*	
H11C	0.7998	0.6214	0.6140	0.056*	
C12	0.4920 (5)	0.67741 (14)	0.5854 (3)	0.0364 (7)	
H12A	0.5209	0.7143	0.6303	0.055*	
H12B	0.3673	0.6769	0.5674	0.055*	
H12C	0.5572	0.6766	0.5096	0.055*	
O3	0.2501 (2)	0.48396 (9)	0.99424 (19)	0.0261 (4)	
H1O3	0.190 (7)	0.503 (2)	1.043 (5)	0.039*	0.50
H2O3	0.316 (7)	0.506 (2)	0.956 (5)	0.039*	0.50
H3O3	0.327 (6)	0.464 (2)	1.026 (6)	0.039*	0.50
H4O3	0.172 (6)	0.464 (2)	0.963 (6)	0.039*	0.50

### Atomic displacement parameters (Å^2^)

**Table:**

	*U*^11^	*U*^22^	*U*^33^	*U*^12^	*U*^13^	*U*^23^
Si1	0.0189 (3)	0.0196 (3)	0.0207 (3)	−0.0005 (3)	−0.0001 (3)	0.0000 (3)
O1	0.0248 (11)	0.0336 (12)	0.0354 (11)	−0.0016 (8)	0.0018 (9)	−0.0151 (9)
C1	0.050 (2)	0.0316 (17)	0.0408 (18)	0.0045 (14)	0.0121 (16)	0.0115 (14)
C2	0.0251 (16)	0.0441 (18)	0.0357 (17)	0.0006 (13)	−0.0040 (13)	−0.0035 (14)
C3	0.0275 (14)	0.0209 (13)	0.0198 (12)	−0.0004 (10)	0.0043 (10)	−0.0001 (10)
C4	0.055 (2)	0.0293 (16)	0.0317 (16)	−0.0003 (14)	0.0047 (15)	−0.0066 (13)
C5	0.077 (3)	0.0299 (17)	0.0342 (17)	0.0152 (17)	0.0179 (18)	0.0108 (14)
C6	0.0287 (17)	0.0419 (18)	0.057 (2)	0.0116 (14)	−0.0020 (15)	−0.0126 (16)
Si2	0.0187 (4)	0.0208 (3)	0.0242 (4)	−0.0002 (3)	−0.0008 (3)	0.0022 (3)
O2	0.0254 (11)	0.0366 (12)	0.0415 (12)	0.0014 (9)	0.0020 (10)	0.0170 (10)
C7	0.053 (2)	0.0360 (17)	0.0315 (16)	−0.0072 (15)	0.0082 (15)	−0.0135 (14)
C8	0.0212 (15)	0.0394 (18)	0.054 (2)	0.0025 (12)	−0.0009 (14)	0.0073 (15)
C9	0.0282 (14)	0.0184 (12)	0.0211 (13)	0.0008 (10)	0.0008 (11)	−0.0017 (10)
C10	0.053 (2)	0.0259 (16)	0.0349 (17)	0.0045 (14)	−0.0021 (15)	−0.0099 (13)
C11	0.0272 (16)	0.0475 (19)	0.0379 (17)	−0.0007 (13)	0.0123 (14)	0.0009 (14)
C12	0.055 (2)	0.0263 (15)	0.0279 (15)	0.0023 (14)	0.0011 (15)	0.0053 (12)
O3	0.0188 (11)	0.0259 (10)	0.0334 (11)	0.0004 (8)	−0.0024 (9)	−0.0003 (9)

### Geometric parameters (Å, °)

**Table:**

Si1—O1	1.651 (2)	Si2—C8	1.859 (3)
Si1—C1	1.852 (3)	Si2—C9	1.871 (3)
Si1—C2	1.853 (3)	O2—H1O2	0.8400
Si1—C3	1.888 (3)	O2—H2O2	0.8400
O1—H1O1	0.8400	C7—H7A	0.9800
O1—H2O1	0.8400	C7—H7B	0.9800
C1—H1A	0.9800	C7—H7C	0.9800
C1—H1B	0.9800	C8—H8A	0.9800
C1—H1C	0.9800	C8—H8B	0.9800
C2—H2A	0.9800	C8—H8C	0.9800
C2—H2B	0.9800	C9—C10	1.528 (4)
C2—H2C	0.9800	C9—C11	1.533 (4)
C3—C5	1.521 (4)	C9—C12	1.535 (4)
C3—C4	1.532 (4)	C10—H10A	0.9800
C3—C6	1.533 (4)	C10—H10B	0.9800
C4—H4A	0.9800	C10—H10C	0.9800
C4—H4B	0.9800	C11—H11A	0.9800
C4—H4C	0.9800	C11—H11B	0.9800
C5—H5A	0.9800	C11—H11C	0.9800
C5—H5B	0.9800	C12—H12A	0.9800
C5—H5C	0.9800	C12—H12B	0.9800
C6—H6A	0.9800	C12—H12C	0.9800
C6—H6B	0.9800	O3—H1O3	0.824 (19)
C6—H6C	0.9800	O3—H2O3	0.82 (2)
Si2—O2	1.648 (2)	O3—H3O3	0.822 (19)
Si2—C7	1.856 (3)	O3—H4O3	0.815 (19)
			
O1—Si1—C1	109.79 (15)	O2—Si2—C9	107.90 (12)
O1—Si1—C2	107.15 (13)	C7—Si2—C9	110.33 (14)
C1—Si1—C2	109.53 (17)	C8—Si2—C9	111.62 (16)
O1—Si1—C3	107.38 (12)	Si2—O2—H1O2	109.5
C1—Si1—C3	110.88 (14)	Si2—O2—H2O2	109.5
C2—Si1—C3	112.00 (14)	Si2—C7—H7A	109.5
Si1—O1—H1O1	109.5	Si2—C7—H7B	109.5
Si1—O1—H2O1	109.5	H7A—C7—H7B	109.5
Si1—C1—H1A	109.5	Si2—C7—H7C	109.5
Si1—C1—H1B	109.5	H7A—C7—H7C	109.5
H1A—C1—H1B	109.5	H7B—C7—H7C	109.5
Si1—C1—H1C	109.5	Si2—C8—H8A	109.5
H1A—C1—H1C	109.5	Si2—C8—H8B	109.5
H1B—C1—H1C	109.5	H8A—C8—H8B	109.5
Si1—C2—H2A	109.5	Si2—C8—H8C	109.5
Si1—C2—H2B	109.5	H8A—C8—H8C	109.5
H2A—C2—H2B	109.5	H8B—C8—H8C	109.5
Si1—C2—H2C	109.5	C10—C9—C11	109.1 (3)
H2A—C2—H2C	109.5	C10—C9—C12	108.6 (3)
H2B—C2—H2C	109.5	C11—C9—C12	109.0 (3)
C5—C3—C4	109.2 (2)	C10—C9—Si2	110.3 (2)
C5—C3—C6	109.4 (3)	C11—C9—Si2	109.2 (2)
C4—C3—C6	108.8 (3)	C12—C9—Si2	110.6 (2)
C5—C3—Si1	110.1 (2)	C9—C10—H10A	109.5
C4—C3—Si1	110.2 (2)	C9—C10—H10B	109.5
C6—C3—Si1	109.13 (19)	H10A—C10—H10B	109.5
C3—C4—H4A	109.5	C9—C10—H10C	109.5
C3—C4—H4B	109.5	H10A—C10—H10C	109.5
H4A—C4—H4B	109.5	H10B—C10—H10C	109.5
C3—C4—H4C	109.5	C9—C11—H11A	109.5
H4A—C4—H4C	109.5	C9—C11—H11B	109.5
H4B—C4—H4C	109.5	H11A—C11—H11B	109.5
C3—C5—H5A	109.5	C9—C11—H11C	109.5
C3—C5—H5B	109.5	H11A—C11—H11C	109.5
H5A—C5—H5B	109.5	H11B—C11—H11C	109.5
C3—C5—H5C	109.5	C9—C12—H12A	109.5
H5A—C5—H5C	109.5	C9—C12—H12B	109.5
H5B—C5—H5C	109.5	H12A—C12—H12B	109.5
C3—C6—H6A	109.5	C9—C12—H12C	109.5
C3—C6—H6B	109.5	H12A—C12—H12C	109.5
H6A—C6—H6B	109.5	H12B—C12—H12C	109.5
C3—C6—H6C	109.5	H1O3—O3—H2O3	113 (4)
H6A—C6—H6C	109.5	H1O3—O3—H3O3	113 (7)
H6B—C6—H6C	109.5	H2O3—O3—H3O3	95 (6)
O2—Si2—C7	109.13 (16)	H1O3—O3—H4O3	98 (7)
O2—Si2—C8	106.94 (13)	H2O3—O3—H4O3	123 (7)
C7—Si2—C8	110.79 (16)	H3O3—O3—H4O3	115 (4)
			
O1—Si1—C3—C5	−60.8 (2)	O2—Si2—C9—C10	59.0 (2)
C1—Si1—C3—C5	179.3 (2)	C7—Si2—C9—C10	178.1 (2)
C2—Si1—C3—C5	56.6 (3)	C8—Si2—C9—C10	−58.3 (2)
O1—Si1—C3—C4	178.8 (2)	O2—Si2—C9—C11	−60.9 (2)
C1—Si1—C3—C4	58.8 (3)	C7—Si2—C9—C11	58.3 (2)
C2—Si1—C3—C4	−63.9 (2)	C8—Si2—C9—C11	−178.1 (2)
O1—Si1—C3—C6	59.4 (2)	O2—Si2—C9—C12	179.1 (2)
C1—Si1—C3—C6	−60.6 (3)	C7—Si2—C9—C12	−61.7 (3)
C2—Si1—C3—C6	176.7 (2)	C8—Si2—C9—C12	61.9 (2)

### Hydrogen-bond geometry (Å, °)

**Table:**

*D*—H···*A*	*D*—H	H···*A*	*D*···*A*	*D*—H···*A*
O1—H1O1···O3^i^	0.84	2.09	2.717 (3)	131
O1—H2O1···O3	0.84	1.96	2.706 (3)	147
O2—H1O2···O3^ii^	0.84	2.04	2.718 (3)	138
O2—H2O2···O3	0.84	2.05	2.707 (3)	135
O3—H1O3···O1	0.824 (19)	1.91 (3)	2.706 (3)	163 (6)
O3—H4O3···O1^i^	0.815 (19)	1.92 (2)	2.717 (3)	164 (6)
O3—H2O3···O2	0.82 (2)	1.89 (2)	2.707 (3)	173 (6)
O3—H3O3···O2^ii^	0.822 (19)	1.92 (2)	2.718 (3)	164 (6)

Symmetry codes: (i) −*x*, −*y*+1, −*z*+2; (ii) −*x*+1, −*y*+1, −*z*+2.
